# *Anaplasma phagocytophilum* Infection in Ticks, China–Russia Border

**DOI:** 10.3201/eid1705.101630

**Published:** 2011-05

**Authors:** Jia-Fu Jiang, Bao-Gui Jiang, Ji-Hong Yu, Wen-Yi Zhang, Hong-Wei Gao, Lin Zhan, Yi Sun, Xiao-Ai Zhang, Pan-He Zhang, Wei Liu, Xiao-Ming Wu, Rong-Man Xu, Wu-Chun Cao

**Affiliations:** Author affiliations: State Key Laboratory of Pathogen and Biosecurity, Beijing, People’s Republic of China;; Institute of Microbiology and Epidemiology, Beijing

**Keywords:** Anaplasma phagocytophilum, ticks, bacteria, China, Russia, border, letter

**To the Editor:** *Anaplasma phagocytophilum,* an emerging human pathogen of public health importance, is transmitted to humans most commonly by tick bites (*1*). The agent has been detected in various species of *Ixodes* ticks around the world (*2*) and in *Dermacentor silvarum* ticks in northeastern People’s Republic of China (*3*), where 3 *A. phagocytophilum* strains were isolated from wild and domestic animals (*4*). In the Asiatic region of Russia adjacent to China, *A. phagocytophilum* was identified in *Ixodes persulcatus* ticks, and *A. bovis* in *Haemaphysalis concinna* ticks (*5*). Human granulocytic anaplasmosis was reported in the southern area of the Russian Far East that borders China (*6*). The objectives of this study were to investigate the prevalence of *A. phagocytophilum* in ticks collected from the China–Russia border and to characterize the agent by molecular biology techniques.

During May–June 2009, host-seeking ticks were collected by flagging vegetation of grassland or woodland along the China–Russia border. Attached ticks were collected from sheep and goats in Hunchun, and from dogs in Suifenhe (Table). All ticks were identified by morphologic features to the species level and the developmental stage by 2 entomologists (Y. Sun and R.-M. Xu). DNA was extracted from tick samples by using Tissue DNA Extract kit (Tiangen Biotechnique Inc., Beijing, China), following the instructions of the manufacturer. Nested PCR was performed to amplify partial citrate synthase gene (*glt*A) of *A. phagocytophilum* as previously described (*7*). To avoid possible contamination, DNA extraction, the reagent setup, amplification, and agarose gel electrophoresis were performed in separate rooms, and negative control samples (distilled water) were included in each amplification.

**Table:** *Anaplasma phgacytophilum* infection in adult ticks from the China–Russia border, 2009*

Survey site	Location	Origin	Tick species, no. positive/no. tested (%)

*Haemaphysalis concinna*	*H.* *longicornis*	*H. japonica*	*Ixodes persulcatus*	*Dermacentor silvarum*	Total
Mohe	52°28.34′N, 123°28.56′E	Woodland	–	–	–	2/49	0/6	2/55 (3.64)
Heihe	52°28.34′N, 123°28.56′E	Grassland	–	–	–	–	0/76	0/76
Jiayin	50°14.19′N, 127°26.39′E	Woodland	2/36	–	–	0/13	0/2	2/51 (3.92)
Xunke	49°34.26′N, 128°28.29′E	Woodland	0/98	–	–	0/3	0/70	0/171
Luobei	48°52.41′N, 130°03.56′E	Woodland	0/50	–	0/19	0/3	2/103	2/175 (1.14)
Tongjiang	47°34.54′N, 130°15.2′E	Woodland	0/4	–	0/20	0/3	0/12	0/39
Huyuan	47°42.42′N, 131°28.37′E	Woodland	1/23	–	–	0/5	–	1/28 (3.57)
Raohe	48°18.05′N, 134°.20.26′E	Woodland	0/30	–	–	4/90	0/4	4/124 (3.22)
HuLin	46°49.48′N, 133°59.11′E	Woodland	0/36	–	–	0/18	4/89	4/143 (2.80)
Mishan	45°50.8′N, 133°09.04′E	Woodland	3/55	–	–	1/29	0/6	4/90 (4.44)
Mudanjiang	45°16.25′N, 131°58′E	Grassland	4/40	–	–	8/120	0/40	12/200 (6.0)
Dongling	44°31.23′N, 130°34.25′E	Woodland	7/261	–	–	1/48	0/44	8/353 (2.27)
Suifenhe	43°53.23′N, 130°46.46′E	Woodland	3/28	–	–	2/53	0/4	5/85 (5.89)
		Dogs	0/52	2/11	–	2/9	–	4/72 (5.56)
Hunchun	44°21.39′N, 130°44.3.18′E	Woodland	23/243	1/148	–	0/9	0/3	24/403 (5.96)
		Goats, sheep	0/4	10/356	–	1/2	0/2	11/364 (3.02)
Total			43/960 (4.48)	13/515 (2.52)	0/39	21/454 (4.63)	6/461 (1.30)	83/2,429 (3.42)

*–, none identified.

*A. phagocytophilum* was detected in 83 of 2,429 adult ticks, with an overall prevalence of 3.42% (Table). The infection rates in the 14 survey sites ranged from 0 to 5.96%, and were significantly different (χ^2^ = 24.43, df = 13; p = 0.027). Except for *H. japonica*, ticks from 4 species, including *H. concinna*, *H. longicornis*, *I. persulcatus*, and *D. silvarum* were found to be naturally infected. The difference in infection rates among tick species was statistically significant (χ^2^ = 13.03, df = 4; p = 0.011). Of 367 attached *H. longicornis* ticks obtained from domestic animals in Hunchun and Suifenhe, 12 (3.27%) were infected with *A. phagocytophilum* (Table). Nymphal ticks were only collected from vegetation in Hunchun, and 30 pools (10 in each pool) of 1,190 *H. concinna* nymphs were positive with an estimated minimum prevalence of 2.52%.

PCR products were purified by TIANgel Mini Purification Kit (Tiangen Biotechnique Inc.) and sequenced. The sequences obtained were compared with previously published sequences deposited in GenBank by using BLAST (http://blast.ncbi.nim.nih.gov/Blast.cgi). The sequences of partial *glt*A from positive samples had 97.1%–100.0% identity in nucleotide sequences and 95.9%–100.0% identity in deduced amino acid sequences, 97.0%–99.4% and 96.5%–99.1% identity to Khabarovsk-01 strain from far eastern Russia (GenBank accession no. AY339602), and 96.1%–97.4% and 93.8%–98.3% identity to other corresponding sequences deposited in GenBank. Eighteen representative variant sequences obtained in this study were included in phylogenetic analysis based on 348-bp nucleotides of *glt*A by using neighbor-joining methods in MEGA 3.0 software (*8*), which found that the *A. phagocytophilum* identified in this study can be placed in a separate clade, together with Russian Khabarovsk-01 strain, which is distinct from previously reported strains from the United States and Europe (Figure A1).

*A. phagocytophilum* infection has been reported in *I. persulcatus* and engorged *D. silvarum* ticks in northeastern China (*3*). In this study, we also found *Haemaphysalis* spp. ticks, including *H. longicornis* and *H. concinna* ticks, to be infected by the agent. This finding indicates that various tick species may be involved in the maintenance and transmission of *A. phagocytophilum*. Both *H. longicornis* and *H. concinna* ticks usually have 3 hosts in their life cycle and can infest a variety of wild and domestic animals such as rodents, deer, scaly anteaters, sheep, goats, and dogs. *Haemaphysalis* ticks are distributed in a broad range of China and sometimes feed on humans. Their competency as a vector for *A. phagocytophilum* and the importance of this agent in public health as well as in veterinary medicine has yet to be investigated, particularly in the areas where they are predominant (*9*). The *gltA* sequence analyses indicated that the agents detected in this study were similar to the strains isolated from rodents and sheep in northeastern China (*4*) and to *A. phagocytophilum* strains from the Russian Far East adjacent to our survey sites. However, the strains from China are genetically distant from *A. phagocytophilum* strains in the United States and Europe. The genetic diversity of *A. phagocytophilum* in various geographic locations deserves further study.

## Figures and Tables

**Figure A1:**
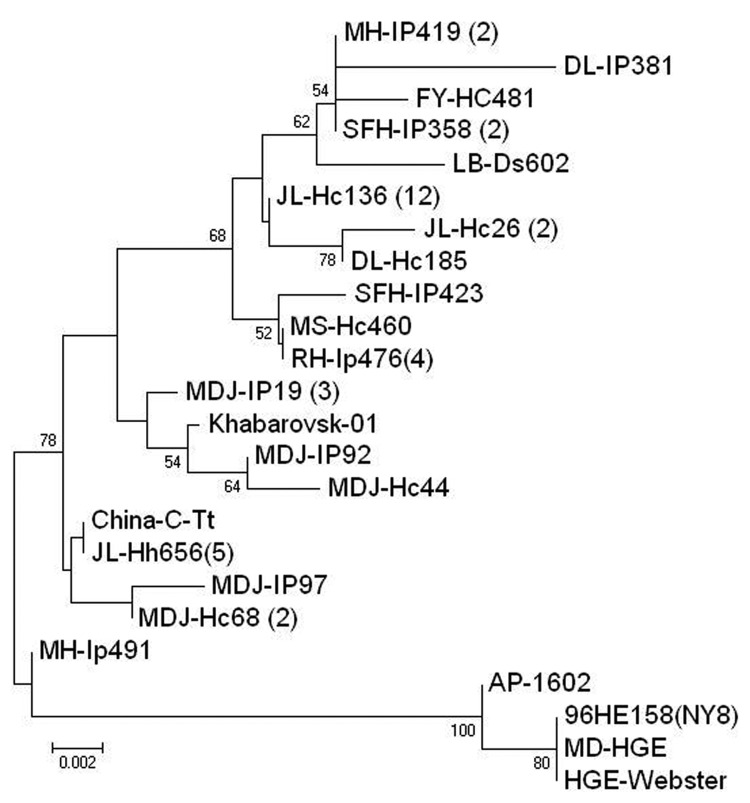
Phylogenetic tree of *Anaplasma phagocytophilum* based on 348-bp the citrate synthase gene. The tree was calculated by neighbor-joining method using MEGA 3.0 software (*8*). Values of the bootstrap support of the particular branching calculated for 10,000 replicates are indicated at the nodes. The 18 variant sequences obtained in this study were designated by the sample site plus vector species and identification number. Scale bar indicates nucleotide substitutions per site. DL: Dongling, FY: Fuyuan, JL: Jilin; LB: Luobei, MH: Mohe, MS: Mishan; RH: Raohe; SFH: Suifenhe; MDJ: Mudanjiang; HC: *Haemaphysalis concinna*, Hh: *H. longicornis*, Ds*: Dermacentor silvarum* IP: *Ixodes persulcatus*. Numbers in parentheses indicates the quantity of the same sequences as this strain. GenBank accession numbers of 42 novel sequences are GU935784–GU935790 and HQ396195–HQ396229. The reference strain were the following: China-C-Tt (GQ412339), 96HE158 (NY8) (AY464136), AP-1602 (AF304138), HGE-Webster (AF304136), MD-HGE(AY464132), and Khabarovsk-01 (AY339602).

## References

[1] Dumler JS, Barbet AF, Bekker CP, Dasch GA, Palmer GH, Ray SC, Reorganization of genera in the families *Rickettsiaceae* and *Anaplasmataceae* in the order Rickettsiales: unification of some species of *Ehrlichia* with *Anaplasma, Cowdria* with *Ehrlichia* and *Ehrlichia* with *Neorickettsia,* descriptions of six new species combinations and designation of *Ehrlichia equi* and “HGE agent” as subjective synonyms of *Ehrlichia phagocytophila.* Int J Syst Evol Microbiol. 2001;51:2145–65. 10.1099/00207713-51-6-2145 11760958

[2] Woldehiwet Z. The natural history of *Anaplasma phagocytophilum.* Vet Parasitol. 2010;167:108–22. 10.1016/j.vetpar.2009.09.013 19811878

[3] Cao WC, Zhan L, He J, Foley JE, De Vlas SJ, Wu XM, Natural *Anaplasma phagocytophilum* infection of ticks and rodents from a forest area of Jilin Province, China. Am J Trop Med Hyg. 2006;75:664–8. 17038691

[4] Zhan L, Cao WC, Jiang JF, Zhang XA, Liu YX, Wu XM, *Anaplasma phagocytophilum* from rodents and sheep, China. Emerg Infect Dis. 2010;16:764–78. 20409364 10.3201/eid1605.091293 PMC2953994

[5] Shpynov S, Fournier PE, Rudakov N, Tarasevich I, Raoult D. Detection of members of the genera *Rickettsia, Anaplasma*, and *Ehrlichia* in ticks collected in the Asiatic part of Russia. Ann N Y Acad Sci. 2006;1078:378–83. 10.1196/annals.1374.075 17114745

[6] Sidelnikov YN, Mediannikov OY, Ivanov LI, Zdanovskaya NI. Clinical and laboratory features of human granulocytic ehrlichiosis in the south of Russian Far East [in Russian]. Epidemiologia i Infectsionnye Bolezni. 2002;3:28–31.

[7] Inokuma H, Brouqui P, Drancourt M, Raoult D. Citrate synthase gene sequence: a new tool for phylogenetic analysis and identification of *Ehrlichia.* J Clin Microbiol. 2001;39:3031–9. 10.1128/JCM.39.9.3031-3039.2001 11526124 PMC88292

[8] Kumar S, Tamura K, Nei M. MEGA3: Integrated software for molecular evolutionary genetics analysis and sequence alignment. Brief Bioinform. 2004;5:150–63. 10.1093/bib/5.2.150 15260895

[9] Teng KF, Jiang ZJ. Economic insect fauna of China. Fasc 39 Acarina: Ixodidae. Beijing [in Chinese]. Beijing:Science Press, Academia Sinica; 1991. p. 359.

